# Under-five mortality in the Democratic Republic of the Congo: secondary analyses of survey and conflict data by province

**DOI:** 10.2471/BLT.22.287915

**Published:** 2022-06-02

**Authors:** Mattias Schedwin, Aurélie Bisumba Furaha, Richard Kapend, Pierre Akilimali, Espoir Bwenge Malembaka, Helena Hildenwall, Tobias Alfvén, Thorkild Tylleskär, Mala Ali Mapatano, Carina King

**Affiliations:** a Department of Global Public Health, Karolinska Institutet, Tomtebogatan 18a, 17177, Stockholm, Sweden.; b Paediatric Department, Hôpital Provincial Général de Référence de Bukavu, Bukavu, Democratic Republic of the Congo.; c School of Criminology and Criminal Justice (SCCJ), University of Portsmouth, Portsmouth, England.; d Kinshasa School of Public Health, University of Kinshasa, Kinshasa, Democratic Republic of the Congo.; e Center for Tropical Diseases and Global Health (CTDGH), Université Catholique de Bukavu, Bukavu, Democratic Republic of the Congo.; f Centre for International Health, University of Bergen, Bergen, Norway.

## Abstract

**Objective:**

To compare coverage of key child health policy indicators across provinces and to explore their association with under-five mortality and level of conflict in the Democratic Republic of the Congo.

**Methods:**

We made a secondary analysis of nationally representative data from 1380 health facilities and 20 792 households in 2017–2018. We analysed provincial-level data on coverage of 23 different indicators for improving common causes of childhood mortality, combined into mean scores for: newborn health, pneumonia, diarrhoea, malaria and safe environment. Using negative binomial regression we compared the scores with provincial-level under-five mortality. With binary logistic regression at the individual level we compared indicators (outcome) with living in a conflict-affected province (exposure).

**Findings:**

All grouped coverage scores demonstrated large ranges across the 26 provinces: newborn health: 20% to 61%; pneumonia: 26% to 86%; diarrhoea: 25% to 63%; malaria: 22% to 53%; and safe environment: 4% to 53%. The diarrhoea score demonstrated the strongest association with under-five mortality (adjusted coefficient: −0.026; 95% confidence interval: −0.045 to −0.007). Conflict-affected provinces had both the highest as well as the lowest mortality rates and indicator coverages. The odds of coverage were higher in conflict-affected provinces for 13 out of 23 indicators, whereas in provinces unaffected by conflict only one indicator had higher odds of coverage.

**Conclusion:**

Conflict alone is a poor predictor for child health. Ensuring that children in unaffected provinces are not neglected while addressing the needs of the most vulnerable in conflict settings is important. Prevent, protect and treat strategies for diarrhoeal disease could help improve equity in child survival.

## Résumé

**Objectif:**

Comparer le degré de couverture des principaux indicateurs propres à la politique de santé infantile dans les provinces, puis examiner leurs liens avec la mortalité chez les moins de cinq ans et le niveau de conflit en République démocratique du Congo.

**Méthodes:**

Nous avons effectué une analyse secondaire des données représentatives à l'échelle nationale provenant de 1380 établissements de santé et 20 792 ménages en 2017–2018. Cette analyse portait sur des données à l'échelle provinciale sur la couverture de 23 indicateurs relatifs à l'amélioration des causes courantes de mortalité infantile, combinées sous forme de scores moyens en matière de santé des nouveau-nés, pneumonie, diarrhée, malaria et environnement sûr. À l'aide d'une régression binomiale négative, nous avons confronté les scores avec la mortalité chez les moins de cinq ans, observée à l'échelle provinciale. Enfin, nous avons utilisé une régression logistique binaire à l'échelle individuelle pour comparer les indicateurs (résultats) avec le fait de vivre dans une province en conflit (exposition).

**Résultats:**

Tous les scores ont montré de grandes disparités entre les 26 provinces: santé des nouveau-nés: 20% à 61%; pneumonie: 26% à 86%; diarrhée: 25% à 63%; malaria: 22% à 53%; et environnement sûr: 4% à 53%. Le score le plus souvent associé à la mortalité chez les moins de cinq ans était celui de la diarrhée (coefficient ajusté: -0,026; IC de 95%: -0,045 à -0,007). Les provinces touchées par des conflits possédaient à la fois les taux les plus bas et les plus élevés en matière de mortalité et de couverture des indicateurs. Les probabilités de couverture étaient plus élevées dans les provinces en conflit pour 13 indicateurs sur 23, tandis que, dans les provinces épargnées, seul un indicateur affichait de meilleures probabilités de couverture.

**Conclusion:**

La santé infantile ne dépend pas uniquement de la présence ou non d'un conflit. Il faut donc impérativement veiller à ce que les enfants des provinces épargnées ne soient pas négligés au moment de répondre aux besoins des plus vulnérables dans les régions en conflit. Des stratégies de prévention, de protection et de traitement contre les maladies diarrhéiques pourraient contribuer à renforcer l'équité dans la survie infantile.

## Resumen

**Objetivo:**

Comparar la cobertura de los indicadores clave de la política de salud infantil entre las provincias y explorar su asociación con la mortalidad de menores de cinco años y el nivel de conflicto en la República Democrática del Congo.

**Métodos:**

Se realizó un análisis secundario de los datos representativos a nivel nacional de 1380 centros de salud y 20 792 hogares entre 2017 y 2018. Se analizaron los datos a nivel provincial sobre la cobertura de 23 indicadores para mejorar las causas comunes de mortalidad infantil, combinados en puntuaciones medias para: la salud del recién nacido, la neumonía, la diarrea, la malaria (o paludismo) y el entorno seguro. Mediante una regresión binomial negativa, se compararon las puntuaciones con la mortalidad de menores de cinco años a nivel provincial. A través de una regresión logística binaria a nivel individual, se compararon los indicadores (desenlace) con el hecho de vivir en una provincia afectada por el conflicto (exposición).

**Resultados:**

Todas las puntuaciones de cobertura agrupadas mostraron grandes rangos en las 26 provincias: salud del recién nacido: 20 % a 61 %; neumonía: 26 % a 86 %; diarrea: 25 % a 63 %; malaria: 22 % a 53 %; y entorno seguro: 4 % a 53 %. La puntuación de la diarrea demostró la mayor asociación con la mortalidad de menores de cinco años (coeficiente ajustado: -0,026; IC del 95 %: -0,045 a -0,007). Las provincias afectadas por el conflicto tenían tanto las tasas de mortalidad como las coberturas de los indicadores más altas y más bajas. Las probabilidades de cobertura eran mayores en las provincias afectadas por el conflicto para 13 de los 23 indicadores, mientras que en las provincias no afectadas por el conflicto solo un indicador tenía mayores probabilidades de cobertura.

**Conclusión:**

El conflicto por sí solo no es un buen factor de predicción de la salud infantil. Es importante asegurar que los niños de las provincias no afectadas no sean desatendidos mientras se atienden las necesidades de los más vulnerables en las situaciones de conflicto. Las estrategias de prevención, protección y tratamiento de las enfermedades diarreicas podrían contribuir a mejorar la equidad en la supervivencia infantil.

## ملخص

**الغرض:**

مقارنة تغطية المؤشرات الرئيسية لسياسة صحة الطفل عبر المقاطعات، واستكشاف ارتباطها بوفيات الأطفال تحت سن الخامسة، ومستوى الصراع في جمهورية الكونغو الديمقراطية.

**الطريقة:**

قمنا بإجراء تحليل ثانوي للبيانات التمثيلية على المستوى الوطني من 1380 مرفقًا صحيًا، و20792 أسرة خلال عامي 2017 و2018. قمنا بتحليل البيانات على مستوى المقاطعات بخصوص تغطية 23 مؤشرًا لتحسين الأسباب الشائعة لوفيات الأطفال، مجمعة في متوسط للدرجات تمثل: صحة الأطفال حديثي الولادة، والالتهاب الرئوي، والإسهال، والملاريا، والبيئة الآمنة. باستخدام الانحدار السلبي ذي الحدين، قمنا بعقد مقارنة بين الدرجات ومعدل وفيات الأطفال تحت سن الخامسة على مستوى المقاطعة. باستخدام الانحدار اللوجستي الثنائي على المستوى الفردي، قمنا بمقارنة المؤشرات (النتيجة) مع الحياة في مقاطعة متأثرة بالصراع (التعرض).

**النتائج:**

أظهرت كل درجات التغطية المجمعة نطاقات كبيرة عبر 26 مقاطعة: صحة الأطفال حديثي الولادة: 20% إلى 61%؛ والالتهاب الرئوي: 26% إلى 86%؛ والإسهال: 25% إلى 63%؛ والملاريا: 22% إلى 53%؛ والبيئة الآمنة: 4% إلى 53%. أظهرت درجة الإسهال أقوى ارتباط مع وفيات الأطفال تحت سن الخامسة (المعامل المعدل: -0.026؛ ‏بفاصل ثقة مقداره 95%: -0.045 إلى -0.007). سجلت المقاطعات المتأثرة بالصراع أعلى وأدنى معدلات للوفيات وتغطية للمؤشرات. كانت احتمالات التغطية أعلى في المقاطعات المتأثرة بالصراع لعدد 13 من أصل 23 مؤشراً، بينما في المقاطعات غير المتأثرة بالصراع كان هناك مؤشر واحد فقط لديه احتمالات أعلى للتغطية.

**الاستنتاج:**

يعتبر الصراع وحده مؤشرًا ضعيفًا على صحة الأطفال. من المهم ضمان عدم إهمال الأطفال في المقاطعات غير المتأثرة أثناء تلبية احتياجات الفئات الأكثر تهميشًا في ظروف الصراع. يمكن أن تساعد استراتيجيات الوقاية والحماية والعلاج الخاصة بمرض الإسهال في تحسين المساواة في الحفاظ على حياة الأطفال.

## 摘要

**目的:**

旨在比较刚果民主共和国不同省份关键儿童健康政策指标的覆盖率，并探讨其与五岁以下幼儿死亡率及冲突水平之间的联系。

**方法:**

我们对 2017 至 2018 年间来自 1380 个卫生机构和 20 792 个家庭的全国代表性数据进行了次级分析。我们分析了 23 个指标覆盖率的省级数据，这些指标用于改善儿童常见的死亡原因，并计算了新生儿健康、肺炎、腹泻、疟疾和安全环境的平均得分。我们使用负二项回归模型比较了省级五岁以下幼儿死亡率数据。通过从个体层面进行二项逻辑回归分析，我们比较了居住在受冲突影响省份（接触）人群的指标（结果）。

**结果:**

全部分组覆盖率得分均显示 26 个省份间存在较大差距：新生儿健康：20% 至 61%；肺炎：26% 至 86%；腹泻：25% 至 63%；疟疾：22% 至 53%；及安全环境：4% 至 53%。腹泻得分显示与五岁以下幼儿死亡率的关联性最大（调整系数：-0.026; 95% CI: -0.045 至 -0.007）。最高和最低死亡率及指标覆盖率均来自于受冲突影响的省份。在受冲突影响省份，23 个指标中 13 个指标的覆盖率均比较高，而在未受到冲突影响的省份，仅有一个指标的覆盖率比较高。

**结论:**

仅冲突这一个因素并不能很好地预测儿童健康。重要的是确保未受冲突影响省份的儿童不会受到忽视并解决冲突地区弱势群体的需求。腹泻疾病的预防、保护和治疗策略有助于提高儿童生存率方面的公平性。

## Резюме

**Цель:**

Сравнить охват ключевых показателей политики в области обеспечения здоровья детей в разных провинциях и изучить их связь со смертностью детей в возрасте до пяти лет и интенсивностью конфликта в Демократической Республике Конго.

**Методы:**

В 2017–2018 гг. авторы провели вторичный анализ общенациональных репрезентативных данных, полученных от 1380 медицинских учреждений и 20 792 домохозяйств. Авторы проанализировали на уровне провинций данные по охвату 23 показателей, применяемых к распространенным причинам детской смертности и объединенных в средние баллы по следующим показателям: здоровье новорожденных, пневмония, диарея, малярия и безопасная среда. Используя отрицательную биномиальную регрессию, авторы сравнили баллы со смертностью детей в возрасте до пяти лет на уровне провинций. С помощью бинарной логистической регрессии на индивидуальном уровне авторы сравнили показатели (исход) с проживанием в затронутой конфликтом провинции (воздействие).

**Результаты:**

Все сгруппированные показатели охвата продемонстрировали широкие диапазоны по 26 провинциям: здоровье новорожденных: от 20 до 61%; пневмония: от 26 до 86%; диарея: от 25 до 63%; малярия: от 22 до 53%; безопасная среда: от 4 до 53%. Показатель диареи продемонстрировал самую сильную связь со смертностью детей в возрасте до пяти лет (скорректированный коэффициент: –0,026; 95%-й ДИ: от –0,045 до –0,007). В провинциях, затронутых конфликтом, были как самые высокие, так и самые низкие показатели смертности и охвата показателей. Шансы охвата были выше в провинциях, затронутых конфликтом, по 13 из 23 показателей, тогда как в провинциях, не затронутых конфликтом, только один показатель имел более высокие шансы охвата.

**Вывод:**

Конфликт сам по себе является плохим предиктором здоровья ребенка. Крайне важно, чтобы дети в провинциях, не затронутых конфликтом, не оставались без внимания во время удовлетворения потребностей наиболее уязвимых лиц в условиях конфликта. Стратегии профилактики, защиты и лечения диарейных заболеваний могут обеспечить справедливость в отношении выживания детей.

## Introduction

The main contributors to mortality in children younger than 5 years in sub-Saharan Africa are lower respiratory infections, diarrhoea, malaria and neonatal conditions,^1^ all of which are targeted by evidence-based global action plans. However, the indicators proposed to track progress by these action plans are commonly only reported on a national level, despite over three quarters of variation in under-five mortality in sub-Saharan Africa being explained by subnational factors.^2^

The Democratic Republic of the Congo accounts for 291 000 (11%) of the 2 766 000 estimated annual deaths in children younger than 5 years in sub-Saharan Africa.^3^ Provincial disparities in under-five mortality have previously been demonstrated,^4^ and still persist.^5^ Previous studies have shown provincial differences in the prevalence of acute respiratory infections, diarrhoea, fever, malnutrition, vaccination coverage and availability of high-quality obstetric care.^6^^–^^9^ However, several of these studies are almost a decade old and only one used the new provincial divisions,^8^ as the country transitioned from 11 to 26 provinces in 2015.

Armed conflicts have generally been associated with a high burden of child mortality and morbidity.^10^ During the Congolese wars, however, post-neonatal mortality increased but neonatal mortality did not.^11^ Additionally, this increased mortality was not found in the post-war period despite the continuing state of conflict.^11^ A recent study demonstrated higher odds of delivery in a health facility but lower access to antenatal services for women in high-intensity conflict areas compared with moderate-intensity conflict areas.^12^ Several studies have acknowledged higher coverage of health services in the eastern provinces, where the conflict is concentrated, hypothesizing that this is due to support from nongovernmental organizations (NGOs) and the United Nations, with donor funding.^4^^,^^13^

We aimed to compare the coverage of key policy indicators for better child health across provinces in the Democratic Republic of the Congo and to explore their association with under-five mortality and level of conflict. A subnational perspective should allow for more targeted roll-out of interventions and health-systems planning to support the country in achieving sustainable development goal (SDG) target 3.2 (to end preventable deaths of newborns and children younger than 5 years) in an equitable way.

## Methods

### Study design

We performed a secondary analysis of data from nationally representative, cross-sectional surveys of health facilities and households in the Democratic Republic of the Congo in 2017–2018. The framework for the study was based on a review of three global action plans to identify key policy indicators for action on common causes of childhood mortality, under the broad themes of prevent, protect and treat.

### Setting

The Democratic Republic of the Congo has an estimated population of 85–100 million^14^^,^^15^ residing across 26 provinces and 516 health zones.^16^ Health care is offered by public and private operators including faith-based organizations.^16^ In addition, several NGOs and international organizations operate in the country.^17^ An estimated 40% of the country’s health-care spending comes from out-of-pocket expenditure, with international donors providing a similar proportion.^18^ Ethical approval for the study was obtained from the Swedish Ethical Review Authority (Dnr 2020–05190).

### Data sources

Data collection and sampling procedures for the data sets have been described elsewhere.^5^^,^^19^^,^^20^ We describe here some important details about the data sets; further details are in the supplementary files in the authors’ data repository.^21^

We obtained data on health indicators and socioeconomic status from two national data sets. The Service and Provision Assessment 2017–2018^19^ used stratified random probability sampling to select 1412 health facilities from a list of all 12 050 operational health facilities, excluding health posts. These facilities were surveyed between October 2017 and April 2018. Of the sampled health facilities, 32 (2.3%) were not surveyed, mainly due to security problems. We extracted data from the inventory section of the data (for example, on medications and equipment), and from the service provider questionnaire (for example on receipt of training in kangaroo mother care).

The Multiple Cluster Indicator Survey 2018 household survey^5^ was designed to provide provincial estimates based on individual-level data using a sample frame based on the 1984 population census. A systematic random sample of 30 households was drawn from each of the 721 clusters giving an overall sample of 21 630 households, of which 20 792 (96.1%) were successfully interviewed between December 2017 and July 2018. Twelve clusters were not visited due to insecurity problems, mainly in Tanganyika and Maniema provinces. We used data from the questionnaires about the household, women and children younger than 5 years. We extracted data on relative socioeconomic status (continuous variable) based on household asset ownership and urban or rural setting.

To obtain data on areas of conflict in the Democratic Republic of the Congo we used a third data set. The Uppsala Conflict Data Program Georeferenced Event Data Set contains global temporally and spatially disaggregated data of conflict events.^22^^–^^25^ For an event to be included it must have resulted in at least one death and the actor involved must have been involved in events that together accumulated to at least 25 deaths in one calendar year. We calculated annual levels of conflict for each province between 2013–2018 to match the time frame used to calculate the under-five mortality. We divided provinces into three different conflict categories, adapting the definition from Uppsala University regarding state-based violence: major conflict (if more than 1000 battle-related deaths had occurred in one of the calendar years), minor conflict (more than 25 battle-related deaths) and no conflict (25 deaths or fewer).^26^

### Data collection

We compiled a list of 47 key policy indicators for action on common causes of childhood mortality from the following documents: (i) Every Newborn action plan;^27^^,^^28^ (ii) Global Action Plan for the Prevention and Control of Pneumonia and Diarrhoea;^29^ and (iii) Global Technical Strategy for Malaria 2016–2030.^30^ We reviewed the national health facility and household surveys for available data on coverage of the identified indicators. We used data on 23 different indicators: 10 of the 15 indicators in the Every Newborn action plan,^27^ 11 of the 18 indicators from the Global Action Plan for the Prevention and Control of Pneumonia and Diarrhoea^29^ and three of the 15 Global Technical Strategy for Malaria 2016–2030 indicators^30^ (Table 1). We excluded indicators if no data were available, the intervention was not implemented at the time of the survey, the indicator was not focused on the child (maternal indicators, for example) or too few observations were recorded. Details about the excluded indicators are in the supplementary files.^32^ We set the target coverage at 80% for all indicators, except exclusive breastfeeding (50%) and caesarean section (10%), using the district-level targets set out by the Global Action Plan for the Prevention and Control of Pneumonia and Diarrhoea and the International Vaccine Access Centre.^31^

**Table 1:** Variables included in the study of under-five mortality and key child health policy indicators, by target condition and outcome, Democratic Republic of the Congo

Indicator and data source	Type of intervention^a^	Action plan definition	Study definition
**Lower newborn deaths to 12 or fewer per 1000 live births by 2030** ^27^			
Exclusive breastfeeding for 6 months^5^	Protect	Percentage of infants aged 0–5 months who are exclusively breastfed	Numerator: No. of children younger than 6 months at the time of the study who were only breastfed in the previous 24 hours Denominator: No. of children below 6 months of age surveyed
Skilled birth attendance^5^	Prevent	Numerator: No. of women aged 15–49 years who were attended by skilled health personnel during their most recent live birth in the 2 years before the survey Denominator: No. of women aged 15–49 years with a live birth in the 2 years before the survey	Numerator: No. of women aged 15–49 years who were attended by skilled health personnel (doctor, nurse, midwife) during their most recent live birth in the 2 years before the survey Denominator: No. of most recent live births among women aged 15–49 in the 2 years before the survey
Early postnatal care contact for infants^5^	Prevent	Numerator: No. of last live births with a postnatal health check in the first 2 days after birth Denominator: Total no. of last live births in the past 2 years	Numerator: No. of last live births in the 2 years before the survey with a postnatal health check in the first 2 days after birth Denominator: No. of last live births in the past 2 years
Kangaroo mother care^19^	Prevent	Numerator: (process indicator) No. of facilities in which a space is identified for kangaroo mother care and where staff have received training in the previous 2 years. Denominator: Total no. of facilities with inpatient maternity services that are assessed	Numerator: No. of health facilities offering childbirth services in which a space was identified for kangaroo mother care and where at least one interviewed health-care worker had received training in the previous 2 years Denominator: No. of health facilities offering childbirth services surveyed
Essential newborn care with early initiation of breastfeeding as tracer indicator^5^	Treat	Numerator: No. of live born infants (in the 2 years before the survey) who are breastfed within 1 hour of birth Denominator: Total no. of live born infants in the 2 years preceding the survey	Numerator: No. of last live born infants (in the 2 years preceding the survey) who were breastfed within 1 hour of birth Denominator: No. of last live born infants in the 2 years preceding the survey
Newborn resuscitation^b,^^19^	Treat	Numerator: (process indicator) No. of facilities with a functional neonatal bag and two masks (sizes 0 and 1) in the labour and delivery service area Denominator: Total no. of facilities with inpatient maternity services that are assessed	Numerator: No. of health facilities that offer childbirth services that had a functioning bag valve mask for neonatal resuscitation Denominator: No. of health facilities surveyed offering childbirth services
Treatment of severe neonatal infection^19^	Treat	Numerator: (process indicator) No. of facilities in which gentamicin is available at suitable peripheral level for treatment of severe neonatal infection Denominator: No. of facilities assessed	Numerator: No. of health facilities offering childbirth services where at least one valid injection bottle of the antibiotic gentamicin was observed the day of the survey Denominator: No. of health facilities offering childbirth services surveyed
Chlorhexidine cord-cleansing^c,^^19^	Treat	Numerator: (process indicator) No. of countries with chlorhexidine on the essential drug list for the purpose of cord-cleansing Denominator: Countries with data from essential medicines list policy	Numerator: No. of health facilities offering childbirth services where chlorhexidine was observed Denominator: No. of health facilities offering childbirth services surveyed
Caesarean section rate^5^	Treat	Numerator: No. of women aged 15–49 years with a live birth in the X years before the survey delivered by caesarean section Denominator: Women aged 15–49 years with a live birth	Numerator: No. of women aged 15–49 years with a last live birth in the 2 years before the survey delivered by caesarean section Denominator: Women aged 15–49 years with a last live birth in the 2 years before the study
Emergency obstetric care^c,^^19^	Treat	Numerator: No. of facilities in the area providing basic or comprehensive emergency obstetric care Denominator: Population of the area (expressed per 500 000 people; note a recent recommendation to use a denominator based on births, not population)	Numerator: No. of health facilities offering childbirth services where all of the following have been performed in the previous 3 months: (i) parenteral administration of antibiotics, (ii) parenteral administration of oxytocic, (iii) parenteral administration of anticonvulsants, (iv) assisted vaginal delivery, (v) manual removal of placenta, (vi) removal of retained products of conception, (vii) neonatal resuscitation Denominator: No. of health facilities offering childbirth services surveyed
**End preventable childhood deaths due to pneumonia and diarrhoea by 2025** ^29^			
Exclusive breastfeeding for 6 months^5^	Protect	Percentage of infants aged 0–5 months who are exclusively breastfed	Numerator: No. of children aged 0–5 months at the time of the study who were only breastfed in the previous 24 hours Denominator: No. of children aged 0–5 months surveyed
Complementary feeding^5^	Protect	Percentage of children aged 6–23 months who received a minimum acceptable diet	Numerator: No. of children aged 6–23 months at the time of the survey who were breastfed and received any type of additional food in the previous 24 hours Denominator: No. of children aged 6–23 months surveyed
Access to improved drinking-water^c,^^5^	Protect	Percentage of households and health-care facilities that report using an improved water source	Numerator: No. of households with access to an improved drinking-water source (piped water, boreholes, tube wells, protected dug wells, protected springs, rainwater and packaged or delivered water) within 30 minutes round trip from premises Denominator: No. of households surveyed (weighted by the no. of household members)
Access to improved sanitation facility^c,^^5^	Protect	Percentage of households and health-care facilities with a hygienic sanitation facility	Numerator: No. of households using improved sanitation facilities (flush toilet, piped water, sewer or septic tank, pit latrine, composting toilet) Denominator: No. of households surveyed (weighted by the no. of household members)
Access to handwashing with soap^c,^^5^	Protect	Percentage of households and health-care facilities with soap and water, and a handwashing facility	Numerator: No. of households with soap and water and a handwashing facility Denominator: No. of households surveyed (weighted by the no. of household members)
Access to clean fuel for cooking^5^	Protect	Percentage of households using clean fuels for cooking	Numerator: No. of households using clean fuels for cooking (electric stove, solar cooking, gas stove, alcohol or ethanol stove) Denominator: No. of households surveyed (weighted by the no. of household members surveyed)
Measles vaccine coverage^5^	Prevent	Percentage of children aged 12–23 months immunized with measles-containing vaccine	Numerator: No. of children aged 12–23 months vaccinated with 1 dose of measles vaccine Denominator: No. of children aged 12–23 months surveyed
Pentavalent vaccine coverage^5^	Prevent	Percentage of children aged 12–23 months who received 3 doses of DTP vaccine	Numerator: No. of children 12–23 months vaccinated with 3 doses of pentavalent vaccine (DTP, Hep B and Hib) Denominator: No. of children aged 12–23 months surveyed
Pneumococcal vaccination coverage^5^	Prevent	Percentage of children aged 12–23 months who received 3 doses of pneumococcal vaccine	Numerator: No. of children aged 12–23 months vaccinated with 3 doses of pneumococcal conjugate vaccine Denominator: No. of children aged 12–23 months surveyed
Oral rehydration therapy^5^	Treat	Percentage of children aged 0–59 months with diarrhoea receiving oral rehydration therapy	Numerator: Children aged 0–59 months with diarrhoea in the 2 weeks before the survey receiving oral rehydration therapy (oral rehydration salt packets) Denominator: No. of children aged 0–59 months with diarrhoea in the 2 weeks before the survey
Zinc for the treatment of diarrhoea^c,d,^^5^	Treat	Percentage of children with diarrhoea who received oral rehydration solutions and an appropriate course of zinc	Numerator: Children aged 0–59 months with diarrhoea receiving zinc in the 2 weeks before the survey Denominator: No. of children aged 0–59 months with diarrhoea in the 2 weeks before the survey
**Reduce burden of malaria by 90% by 2030** ^30^			
Insecticide-treated net^c,^^5^	Protect	Proportion of population at risk who slept under an insecticide-treated net the previous night	Numerator: No. of children younger than 5 years in household who slept under an insecticide-treated net the night before the survey Denominator: No. of children younger than 5 years who slept in their household the night before the survey
Malaria testing^c,^^5^	Treat	Proportion of patients with suspected malaria who receive a parasitological test	Numerator: No. of children younger than 5 years who had fever in the previous 2 weeks who had blood taken from heel or fingertip for testing Denominator: No. of children with fever in the previous 2 weeks
First-line malaria treatment^c,^^5^	Treat	Proportion of patients with confirmed malaria who receive first-line antimalarial treatment according to national policy	Numerator: No. of children younger than 5 years who had fever in the previous 2 weeks and received treatment for malaria (artemisinin-based combination therapy if older than 2 months and quinine if younger than 2 months) Denominator: No. of children younger than 5 years surveyed who had fever in the previous 2 weeks and received treatment for malaria who received any type of antimalarials

DTP: diphtheria–tetanus–pertussis; Hep B: hepatitis B; Hib: Haemophilus influenzae type B.

^a^ Authors’ classification.

^b^ Service and Provision Assessment Survey 2017–2018 does not include a question on mask size.

^c^ Study definition differs from action plan definition.

^d^ We only chose zinc, to be consistent with the international vaccine access centre definition.^31^

Note: Data sources were the Multiple Indicator Cluster Survey, 2017–2018^5^ and Service and Provision Assessment 2017–2018.^19^

We calculated the indicators according to the definitions on Table 1; some indicators were identical to the source reports whereas other differed in definition and were not reported in the reports. We then combined data for the available indicators into six grouped coverage scores covering common causes of childhood mortality, using the same method as the International Vaccine Access Center:^31^ (i) newborn health (using indicators from the Every Newborn action plan); (ii) pneumonia; (iii) diarrhoea; (iv) combined pneumonia and diarrhoea (each from the Global Action Plan for the Prevention and Control of Pneumonia and Diarrhoea); (v) malaria (from the Global Technical Strategy for Malaria 2016–2030); and (vi) safe environment. We generated overall grouped scores by adding the coverage for all included indicators and dividing by the number of indicators in each group (Box 1).

Box 1 **Definitions of grouped scores for child health indicators used in the study of under-five mortality and key child health policy indicators, Democratic Republic of the Congo**

**Newborn health score**

Numerator: exclusive breastfeeding for 6 months, skilled birth attendance, early postnatal care contact for infants, essential newborn care, newborn resuscitation, kangaroo mother care, treatment of severe neonatal infection, chlorhexidine cord-cleansing, caesarean section, emergency obstetric care

Denominator: number of indicators (10)

**Combined pneumonia and diarrhoea score**

Numerator: exclusive breastfeeding for 6 months, pentavalent vaccine coverage, measles vaccine coverage, pneumococcal vaccine coverage, oral rehydration therapy, zinc for the treatment of diarrhoea

Denominator: number of indicators (6)

**Pneumonia score^a^**

Numerator: exclusive breastfeeding for 6 months, pentavalent vaccine coverage, measles vaccine coverage, pneumococcal vaccine coverage

Denominator: number of indicators (4)

**Diarrhoea score^a^**

Numerator: exclusive breastfeeding for 6 months, measles vaccine coverage, oral rehydration therapy, zinc for the treatment of diarrhoea

Denominator: number of indicators (4)

**Malaria score**

Numerator: insecticide-treated net, malaria testing, first-line malaria treatment

Denominator: number of indicators (3)

**Safe environment score**

Numerator: access to improved drinking-water, access to handwashing with soap, access to an improved sanitation facility, access to clean fuel for cooking

Denominator: number of indicators (4)

^a^ We did not include pneumonia care-seeking, pneumonia treatment and rotavirus vaccine coverage due to lack of data.

### Data analysis

Our primary outcome was provincial-level under-five mortality, calculated using the synthetic cohort probability method.^33^ We collapsed the indicator variables to provincial means and summed these into the six indicator grouped scores (Box 1) as the main exposure variables. We applied sample weights to adjust for sampling method for all data taken from the health facility and household data sets. All numerators and denominators presented here are raw data whereas some percentages are weighted. We performed negative binomial regression (due to overdispersion in the data), to estimate the associations between provincial-level under-five mortality and indicator coverage scores for both grouped and individual indicators. Due to collinearity, we analysed each indicator separately.

We adjusted the negative binomial regressions for provincial level of conflict (none, minor or major conflict) and socioeconomic status, reporting the results as an adjusted coefficient. Due to low levels of missing data, we performed a complete case analysis. Differences in mean scores were compared using two-sample *t*-tests.

We performed an individual-level analysis using logistic regression, to explore associations between being covered by an indicator (outcome) and living in a conflict-affected province (exposure), combining major and minor levels of conflict. We adjusted the analysis for household socioeconomic status. The analysis was performed using Stata version 16 (StataCorp, College Station, Texas, United States of America).

## Results

**Fig. 1:**
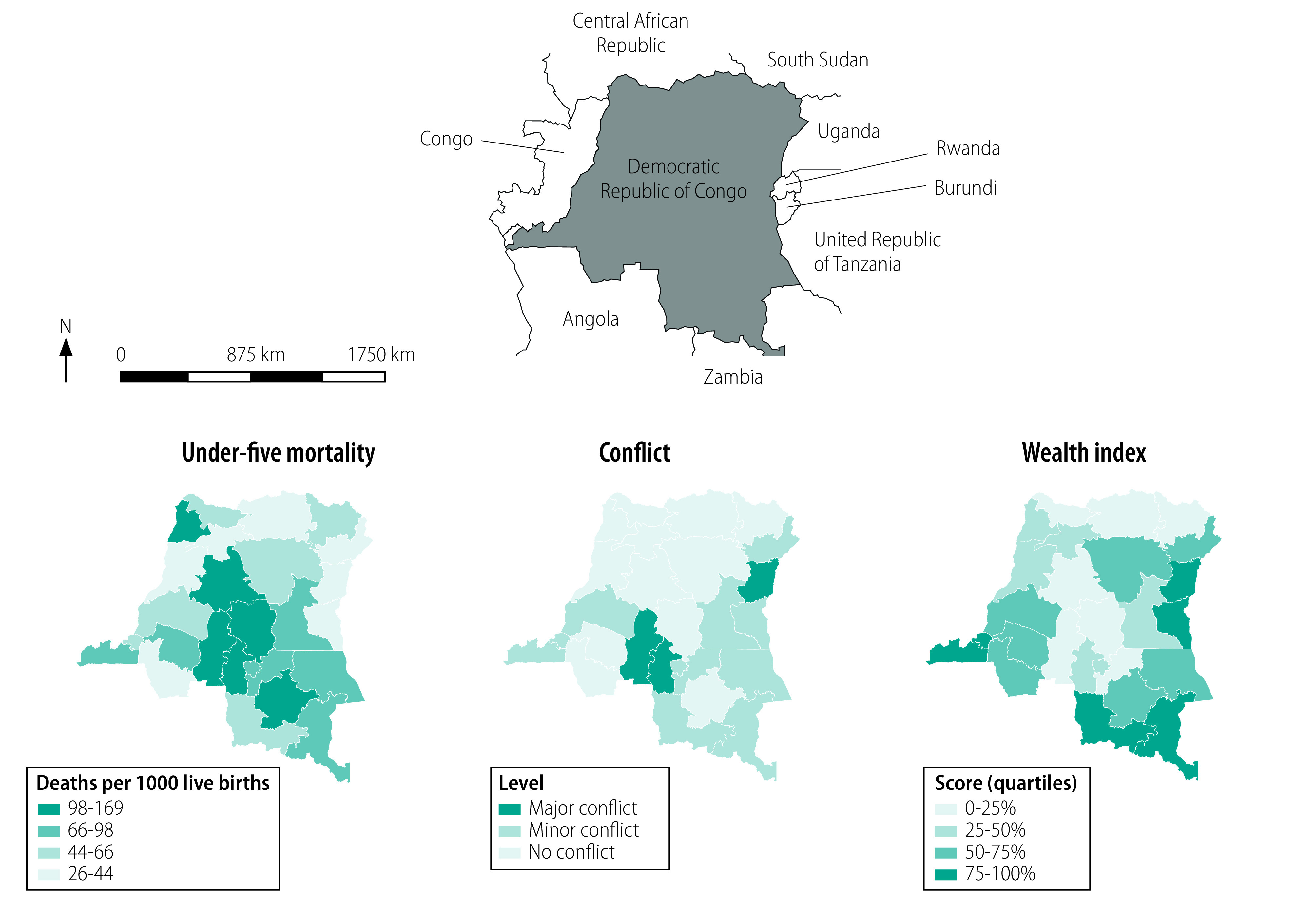
Under-five mortality, conflict level and socioeconomic status (wealth quartiles) by province, Democratic Republic of the Congo, 2017–2018

Overall, there were 1209 under-five deaths among 21 741 reported births. Under-five mortality, socioeconomic status and level of conflict varied considerably across provinces (Fig. 1). Mean provincial socioeconomic status was not significantly associated with under-five mortality (*P* = 0.132). The highest under-five mortality was found in Kasaï (169 deaths per 1000 live births; 95% confidence interval, CI: 134 to 204) and the lowest in North Kivu (26 deaths per 1000 live births; 95% CI: 10 to 42). There were 14 out of 26 provinces classified as conflict-affected, of which three were major conflicts (North Kivu, Kasaï and Kasaï-Central provinces). There were 696 under-five deaths out of the 11 796 reported births among women interviewed in conflict provinces compared with 513 deaths out of 9945 births in non-conflict provinces.

CI: confidence interval.

Notes: Under-five mortality was the number of deaths of infants and children younger than 5 years per 1000 live births. The wealth index is a composite indicator, ranking all included households, using information on ownership of consumer goods and rural/urban status. The wealth index has here been divided into the following wealth quartiles, Q1: 0–25%, Q2: 25–50%, Q3: 50–75%, Q4: 75–100%.

### Indicator coverage

Each indicator showed a considerable range in coverage, with chlorhexidine cord-cleansing having the widest range from 2% in Mongala (6/40 facilities) to 89% in South Kivu (50/59 facilities), followed by pneumococcal conjugate vaccination coverage, ranging from 9% in Sankuru (14/193 facilities) to 90% in North Kivu (129/170 facilities); full data are in the supplementary files.^32^

The target coverage was met on the national level for one indicator, exclusive breastfeeding (median: 54.8%; interquartile range, IQR: 44.6–66.4). However, at the subnational level the target was only met for 16 out of 26 provinces (Table 2). For nine of the 23 indicators, at least one province reached the target coverage. Access to clean fuel for cooking had the lowest coverage at 0% in 16 out of 26 provinces (median: 0.1%; IQR: 0.0 to 0.8), followed by caesarean section (median: 1.8%; IQR: 0.9 to 6.0), access to handwashing with soap (median: 7.3%; IQR: 3.5 to 17.5) and kangaroo mother care (median: 8.1%; IQR: 4.0 to 16.2).

**Table 2:** Median coverage of child health indicators at the provincial level, Democratic Republic of the Congo, 2017–2018

Indicator	Coverage, %			No. of provinces on target (total: 26)	Target, %
	Median (IQR)	Minimum	Maximum		
**Protect indicators**					
Exclusive breastfeeding for 6 months	54.8 (44.6 to 66.4)	30.1	83.7	16	50
Complementary feeding	74.4 (67.2 to 79.0)	58.0	84.7	6	80
Insecticide-treated net	50.2 (40.0 to 66.0)	16.9	75.6	0	80
Access to improved drinking-water	22.4 (10.8 to 34.3)	1.8	74.4	0	80
Access to improved sanitation facility	21.5 (10.4 to 41.1)	0.2	76.8	0	80
Access to handwashing with soap	7.3 (3.5 to 17.5)	0.5	69.9	0	80
Access to clean fuel for cooking	0.1 (0.0 to 0.8)	0	24.5	0	80
**Prevent indicators**					
Skilled birth attendance	78.5 (67.9 to 91.9)	38.4	99.7	12	80
Essential newborn care	43.2 (36.8 to 54.3)	12.1	73.2	0	80
Kangaroo mother care	8.1 (4.0 to 16.2)	0	32.7	0	80
Early postnatal care for infant	50.7 (39.0 to 62.6)	14.1	78.5	0	80
Measles vaccine coverage	44.9 (35.8 to 65.5)	14.8	80.1	1	80
Pentavalent vaccine coverage	34.5 (25.2 to 50.4)	11.1	89.5	1	80
Pneumococcal vaccine coverage	29.8 (22.7 to 51.0)	8.7	89.5	1	80
**Treat indicators**					
Emergency obstetric care	8.8 (4.0 to 14.7)	0	30.0	0	80
Caesarean section	1.8 (0.9 to 6.0)	0	12.3	3	10
Newborn resuscitation	20.6 (11.6 to 40.4)	1.7	48.2	0	80
Chlorhexidine cord-cleansing	41.6 (32.0 to 53.1)	2.0	89.0	2	80
Treatment for severe neonatal infection	68.4 (46.0 to 76.8)	34.8	95.8	3	80
Oral rehydration solution	27.4 (21.0 to 30.9)	7.0	53.4	0	80
Zinc for the treatment of diarrhoea	19.7 (12.0 to 26.9)	4.6	63.2	0	80
Malaria testing	18.2 (12.9 to 22.8)	10.0	45.4	0	80
First-line malaria treatment	37.2 (25.5 to 41.5)	7.5	54.6	0	80

IQR: interquartile range.

### Indicator grouped scores

**Fig. 2:**
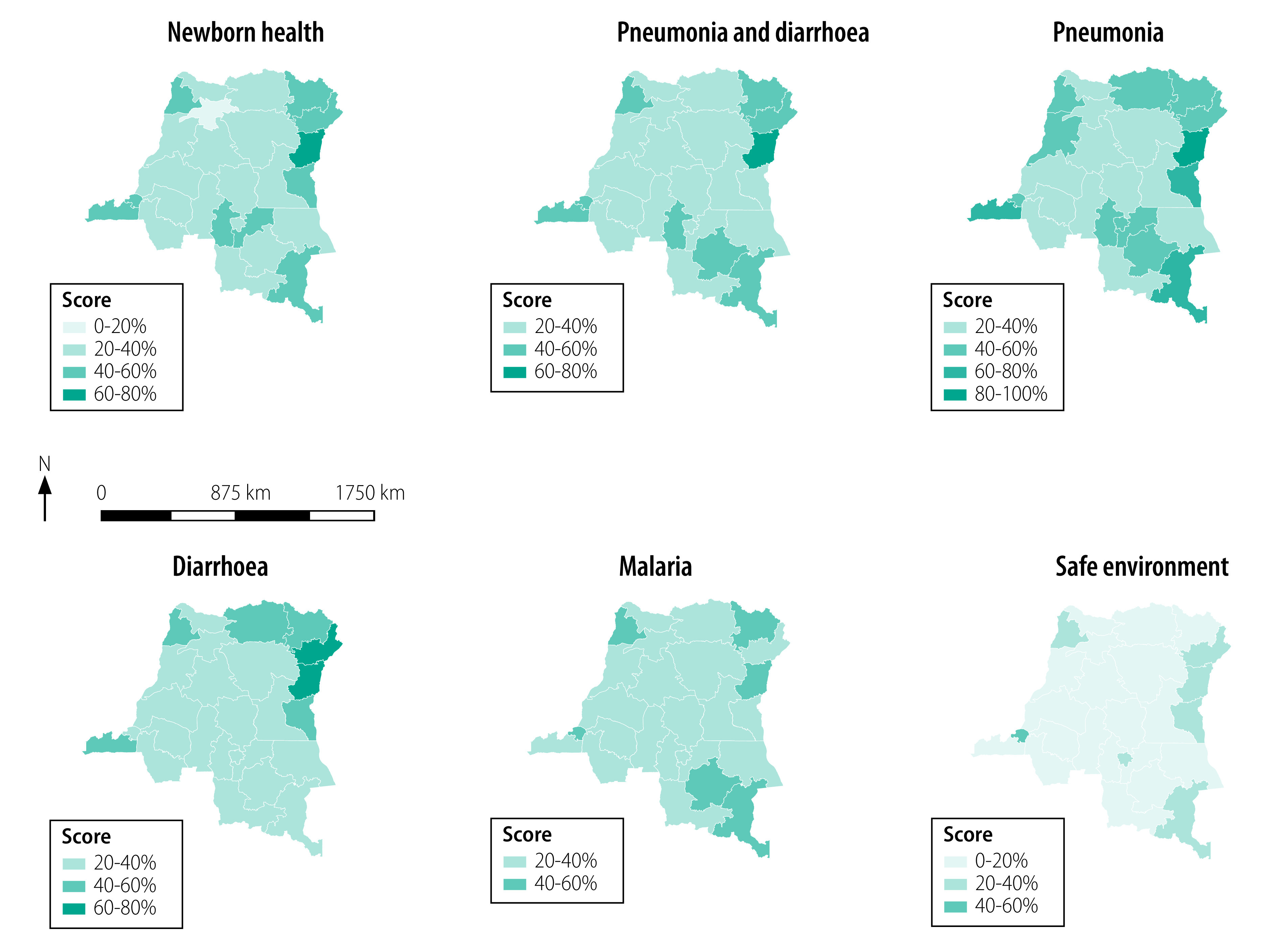
Coverage of grouped indicators for child health by province, Democratic Republic of the Congo, 2017–2018

Note: We calculated grouped indicator scores by summing the coverage for each indicator divided by the total number of indicators in the group.

The national-level overall score on coverage of the 10 indicators for newborn health was 38% (target score: 70%), combined pneumonia and diarrhoea score (6 indicators) was 38% (target score: 75%) and malaria score (3 indicators) was 34% (target score: 80%). These overall scores ranged considerably among provinces for newborn health (Mongala 20%; North Kivu 61%), combined pneumonia and diarrhoea (Kasaï 24%; North Kivu 71%) and malaria (Kwango 22%; Sud-Ubangi 53%; Fig. 2, Table 3). The overall safe environment score (4 indicators) was the lowest, at 17% (target score: 80%), ranging from 4% in Maniema to 53% in Kinshasa.

**Table 3:** Coverage of grouped scores for child health indicators by province, Democratic Republic of the Congo, 2017–2018

Province	Under-five mortality, per 1000 live births	Conflict level^a^	Socioeconomic status, wealth quartile^b^	Grouped indicator scores, %					
				Newborn health (*n* = 10)	Combined and diarrhoea (*n* = 6)	Pneumonia (*n* = 4)	Diarrhoea (*n* = 4)	Malaria (*n* = 3)	Safe environment (*n* = 4)
North Kivu	26	Major	Q4	61	71	86	62	41	31
Kwango	30	No	Q3	34	34	37	36	22	8
Mongala	36	No	Q2	20	26	30	31	38	17
South Kivu	38	Minor	Q4	51	51	62	44	23	23
Bas-Uele	42	No	Q1	40	37	44	41	36	18
Équateur	43	No	Q2	36	39	51	39	33	16
Ituri	44	Minor	Q3	42	59	59	63	26	33
Lualaba	48	Minor	Q4	40	34	38	35	29	15
Nord-Ubangi	53	No	Q1	31	30	39	32	40	11
Haut-Uele	54	No	Q1	49	40	48	44	44	19
Tshopo	60	No	Q3	35	32	34	33	36	18
Kinshasa	60	Minor	Q4	45	46	57	39	41	53
Mai-Ndombe	66	Minor	Q3	37	32	30	38	38	10
Tanganyika	66	Minor	Q3	26	29	35	31	35	19
Kwilu	71	No	Q3	30	29	33	32	35	7
Kongo Central	77	Minor	Q4	46	54	67	46	38	16
Lomami	78	Minor	Q1	41	37	45	37	29	10
Kasaï-Oriental	82	Minor	Q2	40	39	45	37	25	24
Maniema	91	Minor	Q2	34	29	27	39	35	4
Haut-Katanga	98	Minor	Q4	47	42	60	33	43	28
Kasaï-Central	100	Major	Q2	43	44	59	38	34	6
Sud-Ubangi	101	No	Q2	43	40	48	42	53	24
Tshuapa	101	No	Q1	22	28	33	30	32	6
Sankuru	127	No	Q1	36	25	27	33	24	6
Haut Lomami	131	No	Q3	38	42	46	38	41	19
Kasaï	169	Major	Q1	29	24	26	25	23	4
**Overall**	**70**	**NA**	**NA**	**38**	**38**	**45**	**38**	**35**	**17**

NA: not applicable.

^a^ Major conflict: more than 1000 battle-related deaths occurring in one of the calendar years; minor conflict: more than 25 battle-related deaths; no conflict: 25 deaths or fewer.^32^

^b^ The wealth index is a composite indicator, ranking all included households, using information on ownership of consumer goods and rural/urban status. The wealth index has here been divided into the following wealth quartiles, Q1: 0–25%, Q2: 25–50%, Q3: 50–75%, Q4: 75–100%.

Note: We calculated grouped indicator scores by summing the coverage for each indicator divided by the total number of indicators in the group. *n* is the number of indicators in the group. See Box1 for the included indicators. Provinces are sorted from low to high under-five mortality. Data for each individual indicator are in the supplementary files.^21^

### Associations with mortality

Among the overall grouped scores, the diarrhoea score (adjusted coefficient: −0.026; 95% CI: −0.045 to −0.007) and the combined pneumonia and diarrhoea score (adjusted coefficient: −0.019; 95% CI: −0.039 to −0.000) were the only groups with a significant association with under-five mortality; a one-point increase in score resulted in 2.6% and 1.9% fewer deaths per 1000 live births, respectively (Table 4).

**Table 4:** Negative binomial regression of association of grouped scores for child health indicators with under-five mortality, Democratic Republic of the Congo, 2017–2018

Indicator group	Association with under-five mortality				
	Unadjusted coefficient (95% CI)	*r^2^*, %		Adjusted coefficient (95% CI)^a^	*r^2^*, %
Newborn health score	−0.015 (−0.036 to 0.007)	0.7		−0.011 (−0.035 to 0.013)	1.8
Combined pneumonia and diarrhoea score	−0.018 (−0.033 to −0.003)	1.9		−0.019 (−0.039 to −0.000)	2.9
Pneumonia score	−0.012 (−0.023 to −0.000)	1.5		−0.012 (−0.027 to 0.002)	2.5
Diarrhoea score	−0.028 (−0.046 to −0.010)	3.0		−0.026 (−0.045 to −0.007)	3.9
Malaria score	−0.002 (−0.024 to 0.020)	0.0		0.008 (−0.015 to 0.031)	1.7
Safe environment score	−0.014 (−0.028 to 0.000)	1.3		−0.009 (−0.028 to 0.011)	1.8

CI: confidence interval.

^a^ Adjusted for provincial socioeconomic status and conflict levels.

Among the individual indicators for newborn health, caesarean section (adjusted coefficient: −0.083; 95% CI: −0.130 to −0.037) and exclusive breastfeeding (adjusted coefficient: −0.012; 95% CI: −0.022 to −0.001) were significantly associated with decreased under-five mortality (see data repository).^32^ Newborn resuscitation was positively associated with under-five mortality (adjusted coefficient: 0.015; 95% CI: 0.002 to 0.028). Kangaroo mother care (adjusted coefficient: −0.021; 95% CI: −0.043 to 0.001) showed a strong association with mortality but did not meet the significance level. For safe environment indicators, handwashing with soap showed a strong protective association with mortality and was the only statistically significant indicator (adjusted coefficient: −0.016; 95% CI: −0.029 to −0.003). For the pneumonia and diarrhoea indicators, zinc treatment for diarrhoea (adjusted coefficient: −0.009; 95% CI: −0.022 to 0.004) and measles vaccination (adjusted coefficient: −0.008; 95% CI: −0.019 to 0.003) showed the strongest protective association with mortality, but none were statistically significant. No significant correlation was found for the malaria indicators.

### Associations with conflict

Summing the calculated under-five mortality rates for each province divided by the number of provinces, we found that under-five mortality was higher, but not statistically different, in conflict-affected provinces (74 per 1000 live births) compared with provinces unaffected by conflict (71 per 1000 live births, *P* = 0.798). 

**Fig. 3:**
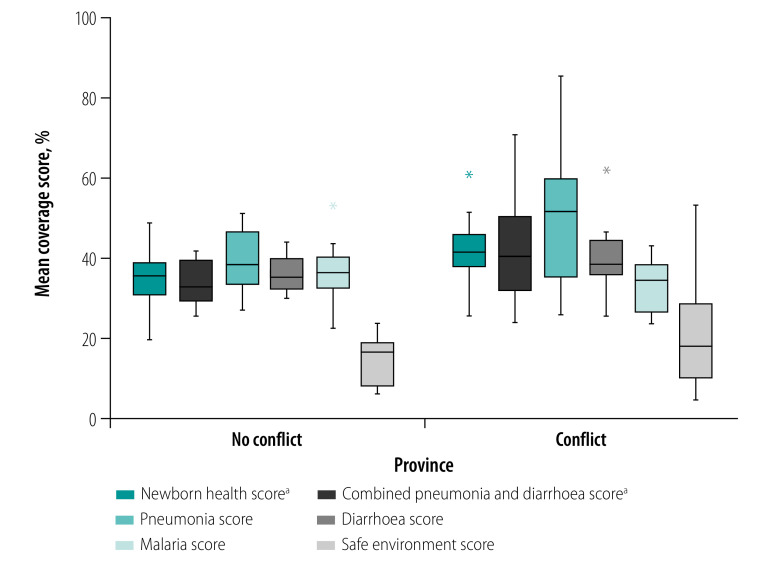
Mean coverage of grouped indicators for child health comparing provinces affected and unaffected by conflict, Democratic Republic of the Congo, 2017–2018

^a^ Statistically significant difference (*P* < 0.05) with two-sample *t*-test.

Notes: Conflict: more than 25 battle-related deaths; no conflict: 25 deaths or fewer.^32^ The boxes in the whisker box plot represent the interquartile range, the whiskers are values within 1.5 times the distance of the interquartile range starting from the limit of the box and the dots are scores further than 1.5 interquartile range from the box limit.

For grouped indicator scores, provinces classified as conflict-affected reported significantly higher mean indicator coverage compared with unaffected provinces for the newborn health score (41%; 95% CI: 36 to 47 versus 34%; 95% CI: 29 to 40, respectively) and for the combined pneumonia and diarrhoea score (42%; 95% CI: 34 to 50; versus 33%; 95% CI: 30 to 37, respectively; Fig. 3).

For 13 out of 23 indicators the odds of coverage of the indicator were higher in conflict-affected provinces. In contrast, only one indicator (sleeping under an insecticide-treated bed net) had higher odds of coverage in a province unaffected by conflict (Table 5). The highest odds of coverage of an indicator in a conflict zone were found for having access to improved drinking-water (adjusted odds ratio, OR: 2.68; 95% CI: 1.90 to 3.78), access to handwashing with soap (adjusted OR: 2.45; 95% CI: 1.67 to 3.60) and receiving pneumococcal vaccine (adjusted OR: 2.42; 95% CI: 1.73 to 3.36).

**Table 5:** Logistic regression comparing coverage of child health indicators (outcome) and living in a conflict-affected province (exposure), Democratic Republic of the Congo, 2017–2018

Indicator	Odds of coverage of indicator in a conflict-affected province	
	Unadjusted OR (95%) CI	Adjusted OR (95% CI)^a^
**Protect indicators**		
Exclusive breastfeeding for 6 months	1.07 (0.80 to 1.43)	1.04 (0.77 to 1.39)
Complementary feeding	0.84 (0.69 to 1.02)	0.88 (0.72 to 1.07)
Access to insecticide-treated net	0.62 (0.51 to 0.76)	0.50 (0.41 to 0.61)
Access to improved drinking-water	3.21 (2.30 to 4.49)	2.68 (1.90 to 3.78)
Access to improved sanitation facility	1.56 (1.18 to 2.08)	1.15 (0.87 to 1.53)
Access to handwashing with soap	3.19 (2.23 to 4.58)	2.45 (1.67 to 3.60)
Access to clean fuel for cooking	2.98 (1.28 to 6.94)	2.14 (0.83 to 5.50)
**Prevent indicators**		
Skilled birth attendance	2.41 (1.67 to 3.46)	1.99 (1.35 to 2.93)
Essential newborn care	1.43 (1.12 to 1.82)	1.42 (1.12 to 1.81)
Kangaroo mother care	2.57 (1.59 to 4.16)	1.71 (1.02 to 2.85)
Early postnatal care for infant	1.52 (1.23 to 1.88)	1.33 (1.08 to 1.65)
Measles vaccine coverage	1.95 (1.46 to 2.62)	1.64 (1.20 to 2.26)
Pentavalent vaccine coverage	2.60 (1.92 to 3.51)	2.23 (1.60 to 3.10)
Pneumococcal vaccine coverage	2.81 (2.07 to 3.80)	2.42 (1.73 to 3.36)
**Treat indicators**		
Emergency obstetric care	2.34 (1.42 to 3.88)	1.71 (0.98 to 2.98)
Caesarean section rate	2.21 (1.36 to 3.58)	2.02 (1.24 to 3.28)
Newborn resuscitation	2.55 (1.78 to 3.66)	1.76 (1.17 to 2.65)
Chlorhexidine cord-cleansing	1.78 (1.31 to 2.42)	1.60 (1.13 to 2.27)
Treatment for severe neonatal infection	1.39 (1.00 to 1.94)	1.17 (0.80 to 1.70)
Oral rehydration solution	0.75 (0.54 to 1.03)	0.72 (0.50 to 1.04)
Zinc for the treatment of diarrhoea	1.39 (0.90 to 2.15)	1.28 (0.79 to 2.07)
Malaria testing	1.65 (1.25 to 2.16)	1.51 (1.13 to 2.01)
First-line malaria treatment	0.95 (0.62 to 1.46)	1.02 (0.64 to 1.62)

CI: confidence interval; OR: odds ratio.

^a^ Adjusted for socioeconomic status.

## Discussion

In our analysis of nationally representative household and facility surveys, we found that target coverage for 14 out of 23 key child health indicators had not been achieved in any province of the Democratic Republic of the Congo. Several of the indicators with the lowest coverage were related to diarrhoea, which also had some of the strongest associations with under-five mortality. Overall, conflict-affected provinces had higher coverage of almost all grouped indicator scores; however, mortality was higher, but not significantly so, in these provinces.

The grouped score for diarrhoea indicators demonstrated the strongest association with under-five mortality, and large disparities in this score were seen across provinces. Diarrhoeal disease remains one of the biggest contributors to under-five mortality, estimated to account for 8% (480 000 deaths) of the 5 300 000 deaths globally^34^ and reported as 9% in the Democratic Republic of the Congo.^35^ Universal coverage with oral rehydration solutions could prevent up to 93% of diarrhoea-related deaths,^36^ but global coverage has remained low at about 42%.^37^^,^^38^ Major improvements can be achieved through increased knowledge about diarrhoea symptoms, availability of oral rehydration solutions and well-trained health-care workers who promote their use.^39^ For the Democratic Republic of the Congo, an important milestone in reducing diarrhoeal disease was the introduction of rotavirus vaccine in 2019, which was not included in our analysis (national coverage was 33% in 2020).^40^ Our results suggest the importance of accelerating access to safe water and sanitation if SDG targets are to be achieved. Access to handwashing with soap had a protective association with under-five mortality in our study and the widest range of coverage between provinces (from 0.5% to 70%). Focusing on relatively low-cost interventions around access to oral rehydration solutions, alongside water, sanitation and hygiene initiatives and equitable vaccine access, could be particularly effective, especially given the country’s high burden of cholera.^41^

Among the neonatal indicators, caesarean section and kangaroo mother care coverage showed the strongest association with under-five mortality. Caesarean section likely reflects the availability of higher-level functional care, but this result should also be interpreted with caution since there are no suggested positive effects on health outcomes with caesarean section rates above 10%.^42^ Kangaroo mother care on the other hand is low-cost and one of the most effective interventions to prevent deaths in low-birth-weight infants.^43^ However, the indicator used in this study showed a low coverage (median 8%, range 0–32%) leaving much room for improvement. Interestingly, researchers found that the quality of maternal and newborn care in North Kivu was low.^44^ In our analysis, however, it was one of the best-performing provinces suggesting that quality improvements are still needed, even when indicator coverage targets are met. Globally, low quality of care is a bigger contributor to mortality than access.^45^ The Democratic Republic of the Congo struggles with medical educational institutions of inadequate quality, a lack of qualified health personnel in general, and a concentration of trained health personnel in the major cities, making high-quality health care challenging.^16^ Poverty and inadequate funding of the health-care sector further complicates accessibility and quality.^16^

Our individual-level analysis showed higher odds of being covered by a policy indicator if the child lived in a conflict-affected province than a province unaffected by conflict. Children in conflict-affected provinces had around double the odds of being covered by several of the water, sanitation and hygiene, vaccination and health-facility indicators. It may be that with long-lasting humanitarian needs and conflict events there is a risk of provinces not affected by conflict being neglected, although this possibility was not raised in the Lancet Series on Women’s and Children’s Health In Conflict Settings.^10^ As an example, South Kivu had the best-funded health system in the Democratic Republic of the Congo in 2012, when taking humanitarian aid into account.^46^ In contrast, mortality was marginally higher in the conflict-affected provinces, although large disparities in mortality were found between conflict-affected provinces. North Kivu had the lowest under-five mortality, highest indicator coverage, and belonged to the highest quartile for socioeconomic status. However, the complete opposite was observed for Kasaï, suggesting that conflict might not be a good predictor of child health or health needs. North Kivu has been affected by conflict since the 1990s, and has a large humanitarian presence,^47^ as compared with Kasaï, which experienced an intense but relatively short conflict episode in the years before data collection. Eastern Democratic Republic of the Congo is also rich in natural resources and has access to cross-border trade, providing the prerequisites for a larger economy that could be a contributor to the higher coverage observed. If targets are to be reached equitably, it is necessary to ensure that well-established patterns of delivering aid do not get in the way of reaching the most vulnerable people.^48^ Our analysis can only report associations, not causation, and therefore it is important that the underlying causes of these disparities are understood and addressed. Furthermore, our provincial analysis does not provide insights into the subprovincial disparities or the children living closest to conflict.^10^

The study had some limitations. First, the ecological approach used for this study only allows for crude analysis and may be limited due to the low number of observations; however, our aim was to give a broad overview of coverage and importance for key child health indicators. The indicators are global targets and, in many ways, act as proxies for a functioning society, infrastructure, health-care systems and political systems. Nonetheless, the strongest associations should be interpreted as potential best-buy interventions to target nationally with a particular focus on the provinces with the lowest coverage. Increasing coverage requires efforts across many sectors, targeting determinants outside the health sector such as poverty, education, food security and good governance,^49^ besides well-trained health-care workers, and increased access to equipment, medication and vaccination,^50^ which are all challenges for the Democratic Republic of the Congo today.

Second, even though Multiple Cluster Indicator Survey data completion rates for major-conflict provinces were high^21^, and the report does not mention any purposeful exclusions due to insecurity, households and facilities in the most insecure areas are likely to have been excluded. The same is likely for households far away from the main roads in the poorest provinces with limited infrastructure. We tried to account for these effects by adjusting for provincial socioeconomic status and conflict level. Additionally, the data do not include children in camps for internally displaced persons or refugees, who constitute a considerable number of children in the Democratic Republic of the Congo.^51^

Third, the sample size did not allow for provincial analysis of all variables, such as care-seeking and treatment for pneumonia. This issue highlights the need for more robust provincial monitoring and evaluation data systems, to improve tracking and data quality. We should also stress that we used multiple hypothesis testing which increases the risk of finding significance by chance. 

Finally, categorizing provinces by conflict intensity level comes with many challenges and, as with any classification approach, important nuances will be missed. Furthermore, the Uppsala University conflict intensity level is intended for state-based violence, whereas we used a broader definition maintaining the same cut-offs.

Our findings suggest that reaching SDG 3.2 is far away for the Democratic Republic of the Congo, and current data indicates that it will not be obtained equitably. Increased efforts are needed in all provinces, and future needs assessments should be based on indicators other than conflict if the equity gap is to be closed.
